# Antimicrobial resistance of *Escherichia coli *isolates from broiler chickens and humans

**DOI:** 10.1186/1746-6148-2-7

**Published:** 2006-02-06

**Authors:** Tricia D Miles, Wayne McLaughlin, Paul D Brown

**Affiliations:** 1 Veterinary Services Division, Ministry of Agriculture, Hope Gardens, Jamaica; 2 Department of Basic Medical Sciences, Biochemistry Section, University of the West Indies, Mona, Kingston 7, Jamaica

## Abstract

**Background:**

Antimicrobial usage is considered the most important factor promoting the emergence, selection and dissemination of antimicrobial-resistant microorganisms in both veterinary and human medicine. The aim of this study was to investigate the prevalence and genetic basis of tetracycline resistance in faecal *Escherichia coli *isolates from healthy broiler chickens and compare these data with isolates obtained from hospitalized patients in Jamaica.

**Results:**

Eighty-two *E. coli *strains isolated from faecal samples of broiler chickens and urine and wound specimens of hospitalized patients were analyzed by agar disc diffusion to determine their susceptibility patterns to 11 antimicrobial agents. Tetracycline resistance determinants were investigated by plasmid profiling, transformations, and amplification of plasmid-borne resistance genes. Tetracycline resistance occurred at a frequency of 82.4% in avian isolates compared to 43.8% in human isolates. In addition, among avian isolates there was a trend towards higher resistance frequencies to kanamycin and nalidixic acid (p < 0.05), while a greater percentage of human isolates were resistant to chloramphenicol and gentamicin (p < 0.05). Multiple drug resistance was found in isolates from both sources and was usually associated with tetracycline resistance. Tetracycline-resistant isolates from both avian and human sources contained one or several plasmids, which were transmissible by transformation of chemically-competent *E. coli*. Tetracycline resistance was mediated by efflux genes *tet*B and/or *tet*D.

**Conclusion:**

The present study highlights the prevalence of multiple drug resistant *E. coli *among healthy broiler chickens in Jamaica, possibly associated with expression of tetracycline resistance. While there did not appear to be a common source for multiple drug resistance in the strains from avian or human origin, the genes encoding resistance are similar. These results suggest that genes are disseminated in the environment and warrant further investigation of the possibility for avian sources acting as reservoirs for tetracycline resistance.

## Background

Antibiotic usage is possibly the most important factor that promotes the emergence, selection and dissemination of antibiotic-resistant microorganisms in both veterinary and human medicine [1,2]. This acquired resistance occurs not only in pathogenic bacteria but also in the endogenous flora of exposed individuals (animals and humans) or populations [3-6]. In intensively reared food animals, antibiotics may be administered to whole flocks rather than individual animals, and antimicrobial agents may be continuously fed to food animals such as broilers and turkeys as antimicrobial growth promoters. Therefore the antibiotic selection pressure for resistance in bacteria in poultry is high and consequently their faecal flora contains a relatively high proportion of resistant bacteria [7].

In Jamaica, chicken is considered to be the most widely produced and consumed meat protein, five times more than beef and 10 times more than pork. Over the period 1998 – 2002, Jamaica's local poultry production yielded approximately 380 M kg with a monetary value of nearly $90 billion [8]. Chicken farms are widely distributed throughout the island with the majority being located on the south coast and large commercial operations accounting for 65–70% of total broiler production [8]. Thus, the high consumption of chicken meat warrants great care in safeguarding the industry against threatening factors.

At slaughter, resistant strains from the gut may contaminate poultry carcasses and as a result poultry meats are often associated with multiresistant *E. coli *[9-11]; likewise eggs become contaminated during laying [12]. Hence, antimicrobial resistant faecal *E. coli *from poultry can infect humans both directly and via food. Although rare, these resistant bacteria may colonize the human intestinal tract and may also contribute resistance genes to human endogenous flora.

However, the mechanism of spread of antibiotic resistance from food animals to humans remains controversial. Colonization of the intestinal tract with resistant *E. coli *from chicken has been shown in human volunteers [13] and there is historical evidence that animals are a reservoir for *E. coli *found in humans [14]. Further, spread of antibiotic resistance plasmids in *E. coli *from chickens to human handlers [15,16] or of antibiotic-resistant microorganisms from poultry to humans in various countries [13,17-19] has been reported. Resistance has been found in organisms common to both humans and animals, such as *Salmonella *spp., *Campylobacter *spp. and enterococci among others [20]. Due to the intricate balance of microflora of different habitats within the ecosystem, the transfer of resistance genes among bacteria occupying different habitats has the potential to occur frequently. Resistance genes may be transferred vertically among bacteria of different genera and families [21] or horizontally among different bacterial species within the same genus or family [22,23].

Molecular tools have been used to correlate animal associated pathogens with similar pathogens affecting humans and to clearly demonstrate transferable resistant genes carried by plasmids common to both animals and humans [2,21,24,25]. The possibility for transfer of antibiotic resistance genes among humans, animals and the environment [22] is a direct threat to public health. This threat prompts ongoing research into emerging resistance mechanisms, novel approaches to antimicrobial efficacy and stringent control measures in the prudent use of antimicrobials in animal and human medicine. As the possibility of transfer of tetracycline-resistant bacteria from animals to humans is controversial, tetracycline-resistant *E. coli *isolates from poultry and humans sources were analysed for plasmid diversity and the presence of specific genes associated with tetracycline resistance.

The aim of this study was to investigate the prevalence and genetic basis of tetracycline resistance in commensal *E. coli *strains isolated from healthy broiler chickens and compare these data with isolates obtained from hospitalized patients.

## Results

### Antimicrobial susceptibility

A total of 82 isolates of *E. coli *were analysed, 34 from broiler chickens and 48 from humans. The percentage of isolates susceptible, intermediate and resistant to each antimicrobial agent is outlined in Table 1. There was a trend towards higher resistance frequency among avian isolates, especially to kanamycin, nalidixic acid and tetracycline (p < 0.05). However, a greater percentage of human isolates were resistant to chloramphenicol and gentamicin (p < 0.05). All avian isolates were susceptible to gentamicin, and there was no significant difference between resistance rates for the β-lactams and the fluoroquinolones for avian and human isolates. No tetracycline-resistant *E. coli *isolate was recovered from feed and water samples from any of the five farms.

Of the aminoglycosides included in the panel, the susceptibility results were varied with 91.2% and 0% of avian *E. coli *isolates expressing resistance to kanamycin and gentamicin respectively. Avian *E. coli *expressed resistance to the β-lactams, ampicillin and amoxicillin/clavulanic acid at frequencies of 20.6% and 2.9% respectively. Tetracycline resistance occurred at a frequency of 82.4%. Among the fluoroquinolones, ciprofloxacin, enrofloxacin, norfloxacin and ofloxacin, resistance expressed by the isolates were relatively similar at 11.8%, 29.4%, 20.6% and 14.7% respectively. Among the avian *E. coli *isolates, frequency of resistance to the first generation quinolone nalidixic acid was approximately four-fold greater when compared with the fluoroquinolones. This difference was statistically significant (p < 0.05).

Most of the human *E. coli *isolates were susceptible to the antimicrobial agents used in the study; and 43.8% and 33.3% showed resistance to the aminoglycosides, kanamycin and gentamicin, respectively, and 43.8% were resistant to tetracycline. An expected difference was observed in the resistance pattern of these isolates to the β-lactams, ampicillin (35.4%) and amoxicillin/clavulanic acid (2.1%). There was a similar low level of resistance to the quinolone family (≤ 10.4%).

**Figure 1:**
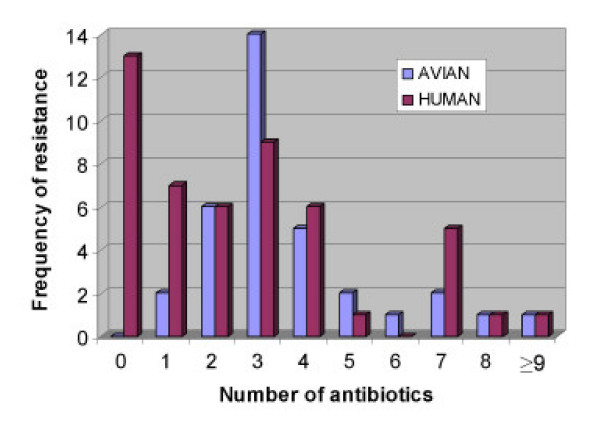
Prevalence of multiple antimicrobial resistance in avian and human *E. coli *isolates.

The prevalence of multiple resistance patterns in avian and human *E. coli *isolates is given in Table 2. The resistance pattern most frequently observed in avian isolates was resistance to kanamycin in combination with nalidixic acid and tetracycline (41.2%). This resistance pattern was observed in avian isolates resistant to four or more antimicrobials and human isolates resistant to seven or more antimicrobials. The next most frequent resistance phenotypes were resistance to kanamycin, nalidixic acid, tetracycline and enrofloxacin at 11.8%, and kanamycin and ampicillin at 8.8%. The remaining 13 avian *E. coli *isolates exhibited a single unique phenotypic pattern and of this, 23% were resistant to 2 antimicrobials, 15.4% were resistant to one antibiotic and 61.5% exhibited unique multiple resistance pattern to ≥ 4 antimicrobials (Table 2; Figure 1). Multi-drug resistance was defined as resistance exhibited to three or more antimicrobials.

Among the human *E. coli *isolates, 72.9% expressed resistance to one or more antimicrobials and 18.8% were resistant to three antimicrobials (Table 2; Figure 1). Approximately 15% of human isolates showed resistance to a single antibiotic (kanamycin, tetracycline or gentamicin), and resistance to two and four antibiotics occurred at a frequency of 12.5% each. Resistance to five or more antibiotics occurred at frequencies of less than 7%.

Isolates that were resistant to other agents were examined for their susceptibilities to tetracycline (Table 3), and isolates resistant to tetracycline were examined for their susceptibilities to other agents (Table 4). Cross-resistance to the various classes of antimicrobials was observed, however, most of these isolates were resistant to tetracycline. Notable exceptions were the ampicillin-resistant avian isolates and the kanamycin-resistant human isolates, of which 42.9% and 37.5%, respectively, were susceptible to tetracycline (Table 3). On the other hand, between 10.4 and 42.4% of tetracycline-resistant (Tet-R) avian isolates were resistant to the fluoroquinolones, in particular enrofloxacin, with almost all being resistant to nalidixic acid and kanamycin (Table 4). Most (96.4%) of the Tet-R human isolates were resistant to ampicillin and kanamycin, followed by chloramphenicol (42.9%), gentamicin (38.1%) and the fluoroquinolones (14.3–23.8%). Although not statistically significant, it was interesting to note that a greater percentage of human isolates were resistant to enrofloxacin.

### Plasmid profiling and transformations

**Figure 2:**
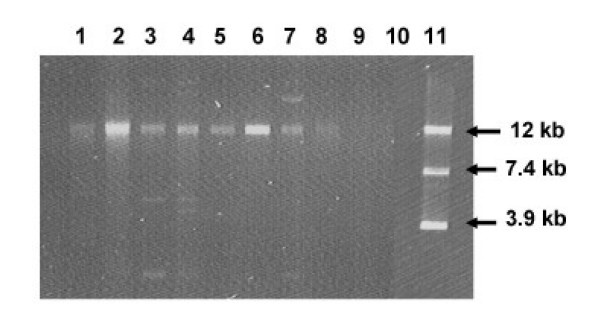
Gel electrophoresis of plasmids isolated from avian and human *E. coli *isolates. Lanes 1, A7, Lane 2, A17, Lane 3, A28, Lane 4, A30, Lane 5, H57, Lane 6, H60, Lanes 7, H61; Lane 8, H78; Lanes 9 and 10, blank; Lane 11, Plasmid markers, pUC4K (3.9 kb), pTYB11 (7.4 kb) and pHS1 (12 kb).

One or more plasmid bands between 2 kb and ≥ 12 kb were observed in all but seven avian and eight human tetracycline-resistant isolates (Figure 2). Defining different profiles as ones differing in at least one plasmid band, six plasmid profiles were observed among the avian isolates and three plasmid profiles were observed among the human isolates.

Purified plasmids from tetracycline resistant isolates were used to transform chemically-competent *E. coli *JM109 (DE3) (Promega), however only the 12 kb plasmid was recovered from transformants. Transformants had similar antimicrobial susceptibilities to donor strains (data not shown).

Digestion of these 12-kb plasmids with *EcoR*1, *BamH*1 and *Hin*d111, did not reveal any significant polymorphisms (data not shown).

### Tetracycline-resistance genes

**Figure 3:**
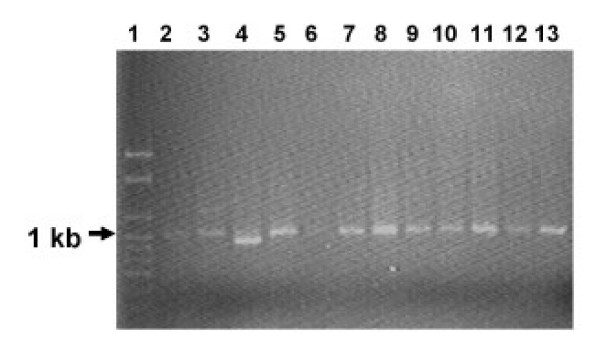
PCR products of tetracycline resistance determinants B and D from tetracycline resistant avian and human *E. coli *isolates. Lane 1, 1 kb ladder; Lane 2, A13; Lane 3, A16; Lane 4, A19; Lane 5, A20; Lane 6, A20T; Lane 7, H60; Lane 8, H62; Lane 9, H71; Lane 10, H97.

Tetracycline-resistance genes of *E. coli *strains expressing tetracycline resistance were amplified in PCR with primers targeting tetracycline efflux genes *tet*B and/or *tet*D. PCR products were obtained for all 21 Tet-R avian and 13 human isolates (Figure 3).

## Discussion

In this study we examined antimicrobial resistance in commensal *E. coli *isolates from broiler chickens and compared them to clinical isolates from hospitalized patients. Because of the geographic sampling of the avian isolates, this surveillance provides a representative sample of the resistance trends in the Jamaican poultry industry.

Compared with the other antimicrobial agents used in this study, avian isolates were susceptible to gentamicin, amoxicillin/clavulanate and chloramphenicol. However, increasing resistance frequencies were noted for the fluoroquinolones, in the apparent absence of selection pressure.

There is strong evidence that the use of antimicrobial agents can lead to the emergence and dissemination of resistant *E. coli *[13,15,16,26-28], which can then be passed onto people via food or through direct contact with animals. However, there are increasing numbers of reports detailing circulation and amplification of antimicrobial resistance genes, including tetracycline resistance in the environment [22,30,29,32], which could facilitate the emergence and spread of antibiotic resistance in bacteria. The finding of multiple drug resistant commensal *E. coli *isolates from broiler chickens from farms that did not report antibiotic use concurs with these latter reports. Rysz and Alvarez [31] demonstrated that bacteria in the soil could acquire resistance to tetracycline from environmental exposure, possibly creating a reservoir of resistance factors generated outside host animals. Their finding suggests that collection of environmental samples from a farm is important for accessing exposure to resistance factors from farm runoff in watersheds, except in cases where soil is augmented with organic fertilizer. Sayah et al. [32] reported that farm environmental isolates showed reduced susceptibility (as measured by disc diffusion zone sizes) compared to faecal sample isolates to most agents studied. They suggested that non-sampled sources, e.g., farm workers and wildlife with access to the farm environment, could be sources of resistance factors. We concur with this as a possible source of resistance determinants in our study.

Antimicrobial resistance to tetracycline, kanamycin and nalidixic acid was noted among avian *E. coli *isolates (Table 1). The presence and frequency of tetracycline resistance in *E. coli *from chickens agree with findings of other studies on antibiotic resistance in *E. coli *[16,32]. The patterns of resistance to tetracycline have been attributed in part to widespread and lengthy use of tetracycline in the poultry industry [3,4,6,15,16]. Since tetracycline is a naturally derived compound, bacteria can be exposed to these agents in nature and outside any human use for disease treatment, for prophylaxis, or for livestock growth promotion. Tetracycline is a commonly used first line antibiotic for many domestic animals and is often used before the antibiotic resistance profile of a pathogen has been determined. Resistance to tetracycline is plasmid mediated, with a wide variety of genetic determinants [33]. This makes it more possible for a susceptible bacterium to acquire resistance factors by conjugation, or by transformation, as was demonstrated in this study.

Low levels of resistance were observed for ofloxacin, ciprofloxacin and gentamicin. The observation that over 85% of avian *E. coli *isolates were resistant to nalidixic acid (concomitant with reduced susceptibilities to the fluoroquinolones) is important considering that the fluoroquinolones are used to treat a range of *E. coli *infections in humans [34]. This finding concurs with previous reports [16,35], and underscores the need to monitor quinolone- and fluoroquinolone-resistant bacteria in poultry production as their emergence is an important health concern among most in the food safety community. This is against the background that nalidixic acid selects for low-level resistance to ciprofloxacin (and possibly other fluoroquinolones) [36]. While quinolone resistance involves chromosomal mutations that reduce membrane permeability and decrease drug accumulation or alter DNA topoisomerases, resistance to fluoroquinolones is most associated with mutations in DNA gyrase [36,37].

Aminoglycoside resistance in *E. coli *most often occurs by aminoglycoside-modifying enzymes [38,39] encoded on transmissible plasmids [40]. It is not surprising that more isolates were resistant to kanamycin when compared to gentamicin, as kanamycin is susceptible to the largest number of enzymes. Conversely, resistance to gentamicin is mediated by modifications at few sites on the molecule.

Multi-drug resistance (to at least three antimicrobials) was found in *E. coli *from both avian and human sources (Table 2; Fig. 1), but was higher in frequency and proportion in avian isolates. However, when these multi-drug resistant organisms were compared, it is clear that they do not have common sources of resistant bacteria. Most avian multi-drug resistant isolates exhibited resistance to a combination of antimicrobials that included kanamycin, tetracycline and nalidixic acid, while most human isolates were resistant to kanamycin and tetracycline or kanamycin and gentamicin. This suggests that avian isolates that were Tet-R are more likely to become resistant to additional antimicrobial agents. Resistance to tetracycline may be conserved in bacterial populations over time, regardless of selection pressure, which might result in an overall increase in resistance over time. In addition, it is likely that avian sources could act as reservoirs for Tet-R for environmental contamination and human colonization. Further, the difference in resistance between the two groups of isolates can be explained in terms of the interactions of the organisms (associated with the host) and potential horizontal gene transfer in their respective environments.

To deal with multi-drug resistant organisms, it is usually recommended that potentially synergistic antimicrobial combinations be used. We evaluated our isolates for cross-resistance between tetracycline and other agents. Isolates resistant to other antimicrobial agents, except ampicillin, were also resistant to tetracycline. Tetracycline-resistant strains from avian sources were susceptible to ampicillin, amoxicillin/clavulanate, chloramphenicol, ciprofloxacin and gentamicin, but resistant to kanamycin and nalidixic acid. This indicates limited cross-resistance and suggests that potential therapeutic options still exist for poultry colonized by Tet-R *E. coli*. On the other hand, a greater percentage of the Tet-R strains from human sources were resistant to other antibiotics, although 57 – 95% was susceptible to amoxicillin/clavulanate, gentamicin and the quinolones. Among these isolates, there was significant cross-resistance to the aminoglycosides, ampicillin, and the quinolones.

Our results indicate that the Tet-R plasmids carried by avian or human *E. coli *isolates demonstrate the distribution of similar resistance determinants in diverse genetic backgrounds. This probably indicates a low level of exposure as animal hosts with a continuous exposure to tetracycline have a higher percentage of Tet-R *E. coli*, and these isolates carry a greater diversity of resistance genes.

In gram negative organisms, including *E. coli*, tetracycline resistance is frequently regulated by efflux genes that are normally associated with large plasmids which are mostly conjugative. These plasmids often carry other antibiotic resistance genes, heavy metal resistance genes and/or other pathogenic factors such as toxins, hence the selection for any of these factors selects for the plasmid. The molecular investigations into the underlying tetracycline resistance mechanisms revealed that 69% of the tetracycline-resistant isolates possessed resistance plasmids based on successful transformations of a susceptible strain. It is likely that the remainder of isolates were using resistance mechanisms based on the chromosome or on integrons carried on plasmids [41,42]. A mechanism for tetracycline resistance was investigated using universal primer pairs based on sequences belonging to the efflux classes, *tetB *and *tetD *[30]. The resulting products of approximately 1 kb would strongly suggest that tetracycline resistance among the *E. coli *isolates in this study is regulated mainly by genes encoding an efflux pump mechanism. These Tet-R strains contained either *tet*B or *tet*D efflux genes, which together with the absence of polymorphism in the plasmid RFLP data, suggest that there is a similarity of tetracycline resistance mechanism in these organisms. Since many human and poultry commensal bacteria carry the same *tet *genes, plasmids, transposons and integrons as disease-causing species [43], our results suggest that these agents could be transferred to disease-causing bacteria.

## Conclusion

In conclusion, the results of this study provide evidence for significant antimicrobial resistance of *E. coli *isolates from broiler chickens raised on farms without recorded antimicrobial use, and hospitalized patients in Jamaica. We noted that tetracycline resistance was generally associated with plasmids, and the resistance was mediated by *tet*B or *te*tD efflux genes. The results suggest that tetracycline resistance mechanisms from avian and human sources may be similar. Long-term prospective studies examining isolates from defined geographic locations are required to more accurately detect temporal and spatial differences in antimicrobial resistance in strains of *E. coli*.

## Methods

### Avian and human *E. coli *isolates

Fresh faecal samples were collected randomly from five poultry farms (approximately 4 km apart) in the parish of St. Catherine, Jamaica between 2000 and 2001. Samples (100 g) were collected over a 42-day period at 3-day intervals, kept at 6°C and bacteriological analyses were performed within 4 h of collection. The flocks investigated, as well as other flocks on the farms consisting of older birds, were not treated with antibiotics during the period of investigation. Farm records indicated that no antimicrobial agents were administered during the 12 months prior to sample collection. Feed and water samples were sampled at each visit to the different farms and screened for *E. coli *resistant to tetracycline. Domestic animals were either absent or located at least 500 m from the broiler operations.

Forty-eight clinical isolates of *E. coli *isolated from urine and wound specimens from hospitalized patients at the University Hospital of the West Indies, St. Andrew, Jamaica during 1999, were also analysed in this study. The University Hospital is a 500-bed, multi-disciplinary teaching hospital that is attached to the Faulty of Medical Sciences at the University of the West Indies in Jamaica. The hospital serves the metropolitan area of Kingston and St. Andrew, and is located about 80 km from the nearest broiler farm that was investigated.

All *E. coli *organisms were isolated and purified on MacConkey agar (Difco laboratories, Detroit, Mich.) and inoculated onto Triple-iron agar, urea agar, Simmons' citrate agar and Motility-indole-lysine media (Difco). Isolates yielding similar biochemical reactions to the standard *E. coli *strain, ATCC 25922 were identified as *E. coli *and selected for further testing. These *E. coli *isolates were transferred to 2 ml Luria broth and incubated 37°C for 18–24 h. One millilitre (1 ml) of this culture was added to 0.8 ml of sterile 80% glycerol in a sterile tube, vortexed and stored at -80°C.

### Antimicrobial susceptibility testing

Antimicrobial susceptibility tests were performed by the standard disc diffusion technique in accordance with the recommendations of the National Committee for Clinical Laboratory Standards (NCCLS) [44]. Antibiotics used in this study are given in Table 1. Cartridges of antimicrobial-containing discs were obtained from Oxoid (Hampshire, UK), Mast Diagnostics (Merseyside, UK), BBL (Becton Dickinson, Cockeysville, MD) or Janssen-Cilag (Puebla, Mexico), stored between 4 and -20°C, and allowed to come to room temperature prior to use. Isolates were subcultured from the bank onto Miller's LB agar and incubated for 18–24 h before being transferred to 5 ml sterile 0.9% saline to match the '0.5' MacFarland standard (Remel, Kansas). A sterile cotton-tipped swab was used to streak air-dried Mueller-Hinton II plates within 15 min of adjustment of turbidity. Subsequently, antimicrobial discs were added and plates were incubated aerobically at 35 ± 2°C for 16–18 h. The diameter of the zones of inhibition surrounding the antimicrobial discs was measured to the nearest mm. Isolates were deemed resistant only when the zone of inhibition was less than or equal to the resistance breakpoint recommended by the NCCLS guidelines [44]. Quality control was performed as recommended using *E. coli *strain ATCC 25922.

### Plasmid profiling and transformations

All isolates resistant to tetracycline (NCCLS interpretive zone ≤ 14 mm) were screened for plasmid content according to the alkaline-lysis mini prep method [45] and resolved on 0.7% agarose gels. Plasmid DNA used as size markers were kind gifts of Dr. S. Morrison. Plasmids were purified from gels and used to transform tetracycline-sensitive chemically-competent *E. coli *JM 109(DE3) (Promega), according to the supplier's instructions. Transformants were analysed on selective media containing 30 or 60 μg/ml tetracycline (Sigma) after incubation at 37°C for 18–24 h. The plasmid-free host strain was included as a susceptible control.

### Restriction fragment length polymorphism (RFLP)

To determine the degree of genetic similarity between resistance plasmids, the 12 kb plasmids were digested with *Bam*H1, *Eco*RI and *Hin*dIII (Promega) according to the manufacturer's instructions. Banding patterns were compared based on visual inspection of the ethidium bromide-stained gels.

### Identification of Tet-R genes

Tetracycline efflux genes (*tetB *and *tetD*) were amplified by PCR with the primers tetBD-F and tetBD-R [30]. The predicted sizes of the PCR-amplified products were 967 or 964 bp for the *tetB *or *tetD *product, respectively. Amplification reactions were carried out in a 25 μl volume containing 5 μl (5 ng/μl) plasmid DNA, 2.5 μl buffer ×10, 2 μl (25 mM) MgCl_2_, 5 μl (500 μM) dNTP mix, 0.25 μl (5 pmol/μl) of each primer, and 0.2 μl (5 U/μl) *Taq *polymerase (Promega). DNA samples were denatured at 95°C for 5 min in a Perkin Elmer GeneAmp System 9600 (PE Biosystems, CA), and amplified by 30 cycles of 94°C for 60 s, 50°C for 45 s, and at 72°C for 90 s, with an additional extension at 72°C for 300 s [30]. Products were separated on 0.7% agarose in 0.5 × TBE and visualized by UV light after staining with ethidium bromide.

### Statistical analysis

The antimicrobial susceptibility data are expressed as percentages or frequency of the avian or human isolates. A one-way analysis of variance or χ^2 ^statistics was used to estimate overall difference between the percentages or frequencies of resistance between avian and human *E. coli *isolates. In all cases, p < 0.05 was regarded as statistically significant.

## List of abbreviations

NCCLS, National Committee for Clinical Laboratory Standards; PCR, polymerase chain reaction; RFLP, restriction fragment length polymorphism; Tet-R, tetracycline resistant.

## Competing interests

The author(s) declare that they have no competing interests.

## Authors' contributions

TDM was involved in the literature search, study design, data collection and interpretation; WM conceived of the study and was involved in study design and data interpretation; PDB was involved in literature search, statistical analysis, data interpretation and manuscript preparation. All authors read and approved the final manuscript.

## Figures and Tables

**Table 1:** Percentages of *E. coli *isolates from avian and human sources susceptible (S), intermediate (I) and resistant (R) to antimicrobial agents by NCCLS disc diffusion methods.

Antimicrobial agent (μg)	Diffusion zone breakpoint (mm)	Avian isolates (n = 34)			Human isolates (n = 48)		
		
		S	I	R	S	I	R
**Aminoglycosides**							
Gentamicin (10)	≤ 12	100	0	0	60.4	6.3	33.3
Kanamycin (30)	≤ 13	2.9	5.9	91.2	8.3	18.8	43.8

**β-lactams**							
Ampicillin (10)	≤ 13	73.5	5.9	20.6	58.3	6.3	35.4
Amoxicillin/clavulanate (20)	≤ 13	85.3	11.8	2.9	79.2	18.8	2.1

**Phenicols**							
Chloramphenicol (30)	≤ 12	88.2	8.8	2.9	68.8	6.3	25

**Quinolones**							
Ciprofloxacin (5)	≤ 15	64.7	26.5	8.8	89.6	0	10.4
Enrofloxacin (5)	≤ 17	32.4	38.2	29.4	89.6	0	10.4
Nalidixic acid (30)	≤ 13	11.8	2.9	85.3	89.6	0	10.4
Norfloxacin (5)	≤ 12	64.7	14.7	20.6	91.7	2.1	6.3
Ofloxacin (5)	≤ 12	85.3	2.9	11.8	91.7	0	8.3

**Tetracyclines**							
Tetracycline (30)	≤ 14	17.6	0	82.4	39.6	16.7	43.8

**Table 2:** Prevalence of multiple resistant patterns in avian and human *E. coli *isolates

Number of antimicrobials resistant	Avian isolates (n = 34)		Human isolates (n = 48)	
	
	Antimicrobial resistance pattern (ARP)	ARP frequency	Antimicrobial resistance pattern (ARP)	ARP frequency
0	-	0	-	13
1	Km; Nal	1;1	Km; Tet; Gen	5;1;1
2	Km-Amp; Km-Nal; Km-Tet; Nal-Tet	3;1;1;1	Km-Gen; Km-Cm; Km-Tet	4;1;1
3	Km-Nal-Tet	14	Km-Gen-Cm; Km-Tet-Amp; Km-Gen-Ofx; Tet-Cm-Amp; Km-Gen-Tet	2;4;1;1;1
4	Km-Nal-Tet-Enr; Km-AMC-Nal-Tet;	4;1	Km-Gen-Tet-Amp; Km-Tet-Cm-Amp	3;3
5	Km-Nal-Tet-Nor-Enr; Km-Nal-Tet-Amp-Nor	1;1	Km-Tet-Cm-Amp-AMC	1
6	Km-Nal-Tet-Ofx-Nor-Enr	1	-	0
7	Km-Nal-Tet-Ofx-Nor-Cip-Enr; Nal-Amp-Tet-Ofx-Nor-Cip-Enr	1;1	Km-Gen-Nal-Tet-Enr-Cm-Amp; Km-Gen-Tet-Cip-Enr-Amp-Cm; Km-Tet-Cip-Enr-Nor-Ofx-Amp; Km-Gen-Nal-Tet-Cip-Cm-Amp; Km-Nal-Tet-Ofx-Nor-Cip-Amp	1;1;1;1;1
8	Km-Nal-Amp-Tet-Ofx-Nor-Cip-Enr	1	Km-Nal-Tet-Cip-Enr-Nor-Ofx-Cm	1
≥9	Km-Nal-Tet-Amp-Ofx-Nor-Cip-Enr-Cm	1	Km-Gen-Amp-Nal-Tet-Cip-Enr-Nor-Ofx	1

Key: Km = kanamycin; Gen = gentamicin; Nal = nalidixic acid; Amp = ampicillin; Tet = tetracycline; AMC = amoxicillin/clavulanate; Cip = ciprofloxacin; Enr = enrofloxacin; Nor = norfloxacin; Ofx = ofloxacin; Cm = chloramphenicol

**Table 3:** Tetracycline susceptibilities of isolates resistant to other antimicrobials

Agent to which isolate was resistant	Avian isolates (n = 34)		Human isolates (n = 48)	
	
	No. of isolates	Isolates susceptible to tetracycline (%)	No. of isolates	Isolates susceptible to tetracycline (%)
Ampicillin	7	3 (42.9)*	17	0 (0.0)
Amoxicillin/clavulanate	1	0 (0.0)	1	0 (0.0)
Chloramphenicol	1	0 (0.0)*	12	3 (25.0)
Ciprofloxacin	4	0 (0.0)	5	0 (0.0)
Enrofloxacin	10	0 (0.0)	5	0 (0.0)
Gentamicin	0	0	16	0 (0.0)
Kanamycin	31	5 (16.1)*	32	12 (37.5)
Nalidixic acid	29	2 (6.9)*	5	0 (0.0)
Norfloxacin	7	0 (0.0)	3	0 (0.0)
Ofloxacin	5	0 (0.0)*	4	1 (25.0)

* p < 0.05 vs. human isolates

**Table 4:** Susceptibilities of tetracycline-resistant isolates to other antibiotics

Antimicrobial agent	Isolates susceptible	
	
	Avian isolates (%) (n = 28)	Human isolates (%) (n = 21)
Ampicillin	24 (85.7)*	4 (19.0)
Amoxicillin/clavulanate	27 (96.4)	20 (95.2)
Chloramphenicol	27 (96.4)*	12 (57.1)
Ciprofloxacin	25 (89.3)	16 (76.2)
Enrofloxacin	16 (57.6)	13 (61.9)
Gentamicin	28 (100.0)*	13 (61.9)
Kanamycin	1 (3.6)	2 (9.5)
Nalidixic acid	1 (3.6)*	16 (76.2)
Norfloxacin	21 (75.0)	18 (85.7)
Ofloxacin	24 (85.7)	18 (85.7)

*p < 0.05 vs. human isolates
